# Undersampled Critical Branching Processes on Small-World and Random Networks Fail to Reproduce the Statistics of Spike Avalanches

**DOI:** 10.1371/journal.pone.0094992

**Published:** 2014-04-21

**Authors:** Tiago L. Ribeiro, Sidarta Ribeiro, Hindiael Belchior, Fábio Caixeta, Mauro Copelli

**Affiliations:** 1 Physics Department, Federal University of Pernambuco (UFPE), Recife, Pernambuco, Brasil; 2 Brain Institute, Federal University of Rio Grande do Norte (UFRN), Natal, Rio Grande do Norte, Brasil; University of Michigan, United States of America

## Abstract

The power-law size distributions obtained experimentally for neuronal avalanches are an important evidence of criticality in the brain. This evidence is supported by the fact that a critical branching process exhibits the same exponent 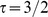. Models at criticality have been employed to mimic avalanche propagation and explain the statistics observed experimentally. However, a crucial aspect of neuronal recordings has been almost completely neglected in the models: undersampling. While in a typical multielectrode array hundreds of neurons are recorded, in the same area of neuronal tissue tens of thousands of neurons can be found. Here we investigate the consequences of undersampling in models with three different topologies (two-dimensional, small-world and random network) and three different dynamical regimes (subcritical, critical and supercritical). We found that undersampling modifies avalanche size distributions, extinguishing the power laws observed in critical systems. Distributions from subcritical systems are also modified, but the shape of the undersampled distributions is more similar to that of a fully sampled system. Undersampled supercritical systems can recover the general characteristics of the fully sampled version, provided that enough neurons are measured. Undersampling in two-dimensional and small-world networks leads to similar effects, while the random network is insensitive to sampling density due to the lack of a well-defined neighborhood. We conjecture that neuronal avalanches recorded from local field potentials avoid undersampling effects due to the nature of this signal, but the same does not hold for spike avalanches. We conclude that undersampled branching-process-like models in these topologies fail to reproduce the statistics of spike avalanches.

## Introduction

Neuronal avalanches are bouts of scale-invariant spatiotemporal electrical activity first recorded by Beggs and Plenz from cortical cultures via multi-electrode arrays (MEAs) [1]. The size 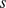 of a neuronal avalanche (defined as the number of active electrodes) is power-law distributed with an exponential cutoff: 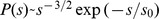, with 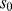 increasing with the number of electrodes of the MEA [1]. The exponent 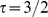 coincides with the mean-field exponent of several classes of models, such as directed percolation and dynamical percolation [2]. In particular, it coincides with the exponent governing a critical branching process [3]. This coincidence has been held as evidence that neuronal avalanches are a statistical signature that the brain as a dynamical system operates near a critical point, a conjecture that has spurred intense research (for recent reviews, see [4] and [5]).

In light of this conjecture, several models for this type of brain activity have been proposed, in which a phase transition occurs between an inactive state and an active collective state. The general idea behind these models is that excitable model neurons can propagate their activity to neighboring model neurons. If coupling is weak enough, any initial activity in the network is bound to die out: the only stable collective state is one of inactivity. However, when coupling is strong enough, activity propagates from neuron to neuron in a never-ending process: self-sustained activity is collectively stable.

A critical point marks the boundary between those two phases. At that point, the theory of critical phenomena predicts that very particular statistical features should appear [6], [7]. For instance, there is no characteristic size for network activity, which will also die out (like in the inactive phase), but without a characteristic time (unlike in the inactive phase). Such lack of characteristic size and time is reflected in power-law event distributions that have been compared with those obtained experimentally also in slices [1], anesthetized rats [8], as well as non-anesthetized resting monkeys [9].

To mimic this critical point, models with very different ingredients have been proposed, such as cellular automata [10]–[14], integrate-and-fire units [15]–[17], or even conductance-based models [18], [19], many of these under diverse underlying topologies [20]–[22]. Networks of excitable cellular automata, in particular, are well-established models allowing simulations of very large system sizes, belonging to the directed percolation universality class [7] and which have been used in direct comparison with experimental results [1], [23]–[25]. For this simple class of models, a very broad class of topologies lead to the same exponent 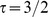 as the classical branching process [3], [26], [27].

Either explicitly or implicitly, the vast majority of these models treat their elementary units as “neurons”. Once the model is tuned to the critical point (or self-organizes itself around it), avalanches are measured by counting the number of “spikes” in those neurons. However, neuronal avalanches are most often measured experimentally from large deviations of local field potentials (LFPs). It is important to emphasize that LFPs sample electrical activity from a radius of up to hundreds of microns, including currents originating from tens to thousands of spiking neurons, as well as from non-spiking, subthreshold neuronal activity [28], [29]. Even non-local contributions are shown to influence LFP measurements [30]. Therefore, when comparing results from these spiking models with experimental data, there has been an implicit assumption in the literature that, at least for the purpose of assessing collective activity at the level of avalanches, LFPs and spikes behave similarly (with the exception of some authors which carefully state that the activity of each of their model units represents the LFP measured at an electrode [25]).

The need for the above mentioned assumption disappears, however, if model results are compared with those obtained from spiking data. In fact, power-law distributed neuronal avalanches of spiking neurons (instead of LFP activity) were experimentally observed in intact leech ganglia [31], dissociated cultures of rat hippocampal [31] and cortical [32] cells, as well as in the primary sensory neocortices of anesthetized rats [33]. In the *in vitro* experiments, the same exponent 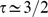 was observed [31], [32], whereas in the anesthetized rat the exponent was in the range 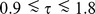. Given the plausibility of branching-process-like models in mimicking the transmission of spikes across neurons and the power-law size distribution they produce at their critical parameter, one could argue that they are a successful minimal theory of spike avalanches.

Despite this apparent success in reproducing the experimental results, however, one crucial aspect which has been almost completely neglected in the models is undersampling: while a typical 32-electrode MEA can record spikes from about 30–100 neurons in an area of about 1–2 mm^2^ of brain tissue, 1 mm^3^ of mammalian cortex comprises on the order of 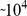 neurons [34]. For models to be adequately compared with experimental results, this fact should be taken into account. Note that this is a completely different problem from what is known in the statistical physics literature as finite size scaling (FSS) [7]. FSS amounts to observing how results change as the model system size increases, *while recording from all sites*. What we propose here is quite different: we simulate large system sizes (mimicking the fact that the brain comprises a huge number of neurons), but measure avalanches only in a subset of the units (mimicking the fact that MEAs record only from a very small fraction thereof).

In the few models which tackled this issue, undersampling was shown to affect the avalanche size distributions observed in critical systems. Priesemann et al. [35], [36] have focused on classical models of the statistical physics literature which exhibit Self-Organized Criticality (SOC), such as the Bak-Tang-Wiesenfeld sand-pile model [37] and variants thereof as well as the Drossel-Schwabl forest-fire model [35], [38]. We have previously employed networks of excitable cellular automata [33], [39] whereas Girardi-Schappo et al. have simulated lattices of coupled maps [40], both of which could be tuned to the critical point.

These lines of research have shown that, when undersampled, these critical-by-construction models yield size distributions which are not necessarily power laws. For instance, we have shown that, when undersampled, excitable cellular automaton models yield size distributions which are very well fit by lognormal functions, in remarkable similarity to data obtained from freely-behaving animals [33]. In this case, therefore, undersampling could reconcile the hypothesis of an underlying critical system with non-power-law experimental results. While it solves one problem, however, it creates another.

Anesthetized animals as well as in vitro preparations do yield spike avalanches whose size distributions are well fit by power laws [31]–[33]. And these are measured with the same MEAs, therefore subjected to the same undersampling conditions. But if undersampled models yield non-power-law distributions, can they be reconciled with these spiking data?

The main purpose of this paper is to systematically probe what can be considered the theoretical workhorse in the field of neuronal avalanches, namely branching-process-like models at their critical points. Specifically, we investigate whether power-law distributions emerge when activity from networks with different topologies is measured only from a subset of their model neurons, in a MEA-like configuration. We screened parameter space exhaustively, changing both the dynamical regime of the system (subcritical, critical, supercritical) as well as the extent of the undersampling (size and density of the model MEA). We also compared the distributions obtained through the model to those obtained experimentally from anesthetized rats.

## Results

We have simulated networks of excitable neurons modelled by cellular automata (see Methods). An 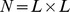 two-dimensional array of model neurons was connected following two rules: 1) a local rule, in which each neuron sends 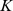 synapses to neighbor neurons at a distance 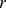 with probability 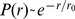 (where 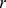 is the distance measured in lattice units, i.e. cell bodies); 2) a non-local rule, in which each of the 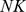 synapses can be rewired to a randomly chosen neuron with probability 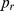. The emergent features of the resulting topology depend on 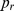.

**Figure 1:**
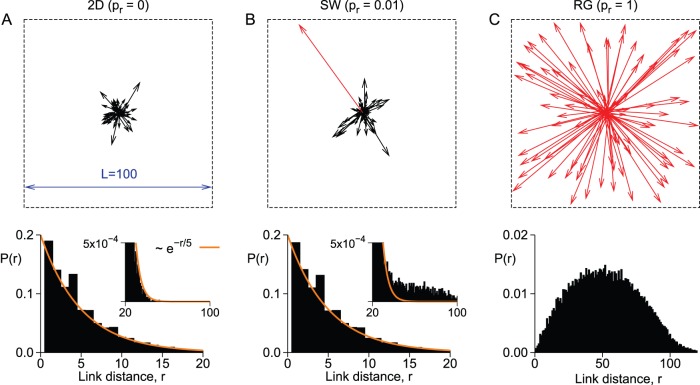
Network topology. Examples of synaptic reach (top) as well as the link size distribution 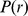 (bottom) for the: A) two-dimensional network; B) small-world network; C) random network. Red arrows in top panels represent synapses which have been rewired (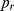 is the rewiring probability). Dashed lines represent the network limits.

For 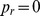, the network is essentially two-dimensional (when 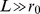). In this case, each site has a well-defined neighborhood and, for large 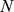, the mean distance between sites increases as 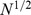. We refer to these networks as two-dimensional (2D). For 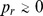, a small-world network (SW) is observed. While a well-defined neighborhood is present (like in the 2D network), there are also long-ranged connections (unlike in the 2D network). For large 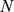, the mean distance between sites increases as 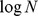 [41]. For 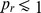, the network is random (RG). In this case, the concept of neighborhood is meaningless, with each site sending its post-synaptic connections to randomly chosen sites across the network. The mean distance between sites, as in the small-world network, also increases as 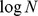. A general picture of these topologies can be seen in Figure 1. Panels A, B, and C (top) show the outgoing synapses from five sites at the center of a 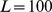 network with 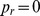, 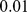 and 1, respectively. The red arrows indicate links which have been rewired. At the bottom, the distributions of link distances are shown. Although the difference between the two-dimensional and the small-world networks seems tiny (note the very small difference in the amount of large links in the insets), the critical exponents observed in the SW network at criticality put it in the same universality class as the random network.

As discussed before, each active site has a chance of propagating the spike to its post-synaptic sites. The transmission probability per link, 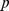, is the control parameter of this model. The next step is, therefore, to find the values of 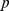 in which each of those network topologies are at their critical points.

### Determining Criticality in the Model

**Figure 2:**
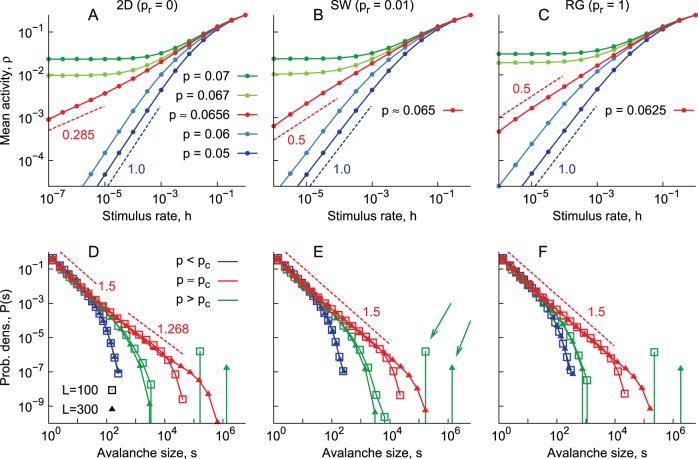
Determining the critical point. A) Response curves for the two-dimensional network, with varying transmission probability 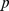. Blue curves are obtained from subcritical systems, with the dashed blue line indicating a 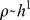 relationship. Green curves are obtained from supercritical systems, whereas the red one is obtained from a critical system. The red dashed line indicates a 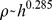 relationship. B) Same as in A, but for a small-world network. The red dashed line here indicates a 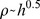 relationship. C) Same as in B, but for a random network. D) Avalanche size distributions for the two-dimensional network. Different 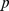 (same color code as in top panels) and system sizes 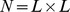 (symbols) are shown. Dashed lines represent exponents of 1.5 (top line) and 1.268 (bottom line). E) Same as in D, but for the small-world network. The dashed line represents an exponent of 1.5. Green arrows highlight the infinite avalanches observed in supercritical systems (see Results). F) Same as in E, but for the random network.

In order to determine the critical point for each topology used, we measured how the mean density of active sites 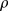 (the order parameter of this model) depends on the Poissonian rate of external stimulus 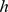 (see Methods). The response curves 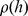 can be seen in Figure 2 (panels A, B and C).

Independently of the underlying topology, when 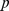 is low enough, once a site is activated by the external stimulus, the activity does not propagate too far. For each incoming stimulus, a characteristic number of spikes will be generated. In this case, therefore, for 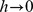, the response scales linearly, 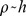 (see the blue curves in Figs. 2A, B and C). This is the subcritical regime.

When 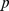 is large enough, the activity is amplified to an extent that external stimuli is no longer needed to maintain neurons spiking: self-sustained activity becomes stable. For 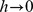, therefore, the response 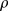 converges to a nonzero value (see the green curves in Figs. 2A, B and C). This is the supercritical regime.

At the critical point there is no self-sustained activity but, since the system is governed by fluctuations, there is no longer a characteristic number of spikes generated by each incoming external stimulus. Therefore, unlike the subcritical case, the response function is no longer linear at criticality, and one expects instead 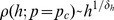, where 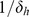 is a critical exponent [6]. Our model is known to belong to the universality class of the directed percolation model [21], [42] (i.e. both models have the same set of critical exponents [6]). For two-dimensional networks, the expected result for this universality class is 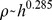 [2], [7], which is confirmed in Fig. 2A (red curve). For both the small-world and random networks, we recover the mean-field result 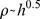 [2], [7] (red curves in Figs. 2B and C).

What are the effects of the subcritical, critical and supercritical regimes on the avalanche size distributions? Avalanches are created by firing the central neuron of a quiescent network, their size being defined as the number of spikes that occurred until the network returns to rest (see Methods). This corresponds to the limit 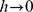 of infinite separation of time scales, in which avalanches do not overlap (in contrast to, say, the situation in Figs. 2A, B and C).

For subcritical systems, short-tailed curves are obtained, with avalanche characteristic sizes independent of the network size (blue curves in Figs. 2D, E and F). For supercritical systems, a finite fraction of the avalanches propagate indefinitely. Since in the simulations we set a maximum time for avalanche spreading (see Methods), these infinite avalanches contribute to the high-value bumps in the size distributions (see the green arrows in Fig. 2E, for example).

At the critical point, avalanche size distributions follow power laws with well-defined exponents. Once again in agreement with the literature [2], [7], mean-field exponents were obtained for small-world and random networks, 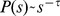, with 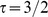 (as represented by the dashed lines in Figs. 2E and F). For the two-dimensional network a crossover between two regimes was observed. For the larger avalanches the 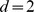 exponent was obtained (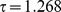), while the size distribution for the smaller avalanches was well fit by the mean-field exponent (Fig. 2D). The explanation of this phenomenon is straightforward: since link distances are exponentially distributed with a characteristic value of 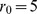 lattice sites, but are otherwise unstructured, small avalanches (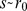) propagate as if they were in a small-world-like network (in the sense that, at that scale, there is a well-defined neighborhood, but also exponentially rare shortcuts to more distant sites). As for the avalanches that keep spreading and become large enough, the range 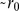 of interaction among neurons is much less than the avalanche size 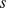. At the large scale, interactions become effectively local, and the governing dynamics is that of a two-dimensional network.

### Size Distributions of Undersampled Model Avalanches

To check whether those power laws persist when the system is not completely sampled, or if non-power-law size distributions observed in subcritical and supercritical systems can turn into power laws under certain sampling configurations, we implemented a sampling matrix mimicking the MEAs employed in extracellular recordings.

**Figure 3:**
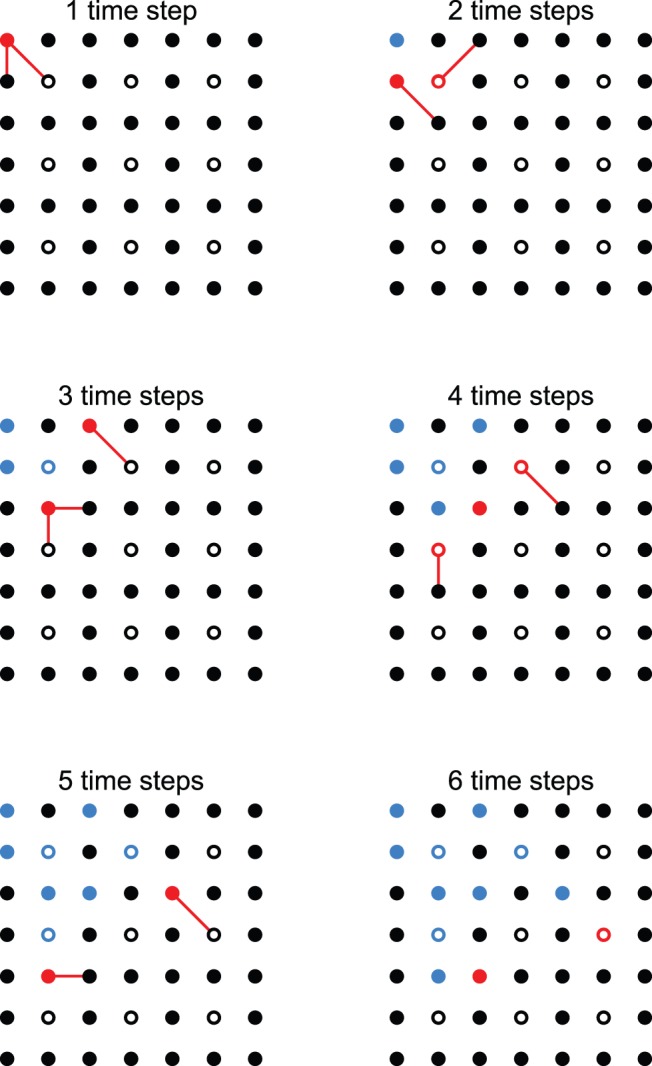
Avalanche propagation. Example of one avalanche propagating in an undersampled 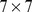 network for 6 time steps. The 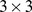 sampling matrix is denoted by empty circles. Red circles represent active sites, blue indicates sites that were activated during avalanche propagation. Red lines show spike propagation.

The sampling matrix is a square 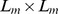 array (centered in the network) of “virtual electrodes”, with a distance 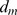 between electrodes. As in the experimental MEAs, each electrode can measure the spiking activity from zero up to 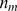 neighboring sites. We investigated how avalanche size distributions change when 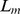 and 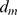 vary. For these simulations, avalanches are created by activating the site at the center of the network, and letting the avalanche spread until it dies out. However, due to the fact that we are not “measuring” at all sites, the activity generated by that single initial excitation may be “read” as a smaller avalanche, or even a series of smaller avalanches. Consider, for instance, the example of Fig. 3, which for simplicity depicts a 2D network of 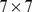 model neurons connected to their nearest and next-nearest neighbors. The activity initiated at the top left site propagates during 6 time steps, which would be the duration of the avalanche if all sites were sampled. During this avalanche 12 spikes occurred, so 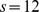 would be the size of the avalanche if all sites were sampled. Note, however, that if we were to assess the network activity from what is measured in the 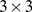 sampling matrix (empty circles in Fig. 3), 1 spike would be measured at the second time step, followed by one time step of silence, which would be interpreted as the end of an avalanche. Then two avalanches would follow, of sizes 2 and 1. The three avalanches detected would all have duration of 1 time step. The question then is how the statistics of avalanche size, which are well known for fully sampled systems, are affected by undersampling.

**Figure 4:**
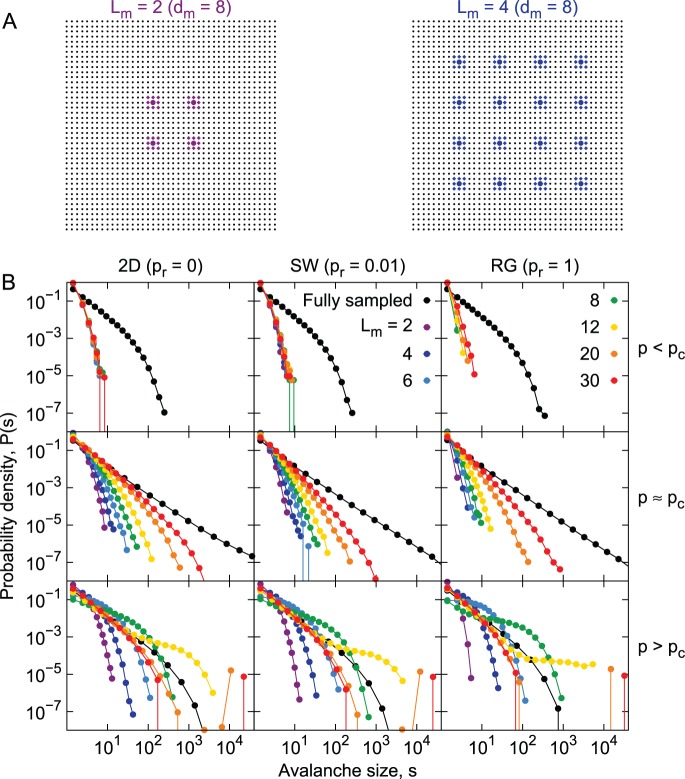
Undersampled size distributions: varying the number of sampled sites. A) Examples of the sampling matrix. Only the colored sites are considered for avalanche measuring. Colored circles indicate virtual electrodes center, with diamond indicating possible sampling sites (see Methods). B) Avalanche size distributions obtained from different underlying topologies (columns) and dynamical regimes (rows), while sampling the systems with a MEA-like configuration. Colors represent different sizes of the virtual sampling MEA (black for fully sampled systems).

We started by investigating the situation in which the distance between electrodes was fixed, and varied the number of electrodes (Figure 4A). Virtual electrodes were set apart by 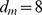 lattice sites (i.e. cell bodies), which corresponds roughly to the 250 µm distance among electrodes in a typical MEA. The size distributions are shown in Figure 4B for the three network topologies considered, as well as the three dynamical regimes (subcritical, critical and supercritical).

For the subcritical systems (top row of Fig. 4B), the size distributions do not change significantly as 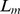 increases. This is expected, since in this case avalanches are unlikely to travel much farther than a characteristic distance. However, in the RG, we do see a decrease in the probability of observing large avalanches as the sampling matrix gets smaller. This is also expected. Due to the lack of a well-defined neighborhood, avalanches will often spread to sites distant from the sampled ones. This becomes more frequent as the number of sampled sites decreases.

Avalanche size distributions in undersampled supercritical systems (Fig. 4B, bottom row) behave exactly as the fully sampled system when 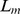 is large enough: there is a fast decrease in the probability of measuring avalanches of size 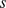, as 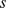 increases, and there is a fraction of the avalanches which will propagate indefinitely. In critical systems (Fig. 4B, middle row), size distributions slowly become more heavy-tailed as 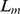 increases. However, the power-law shape is not recovered, even for sampling matrices with a number of electrodes much larger than what is employed experimentally (note that 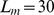 corresponds to 900 electrodes, which is near the state of the art of multi-electrode recordings [43]).

**Figure 5:**
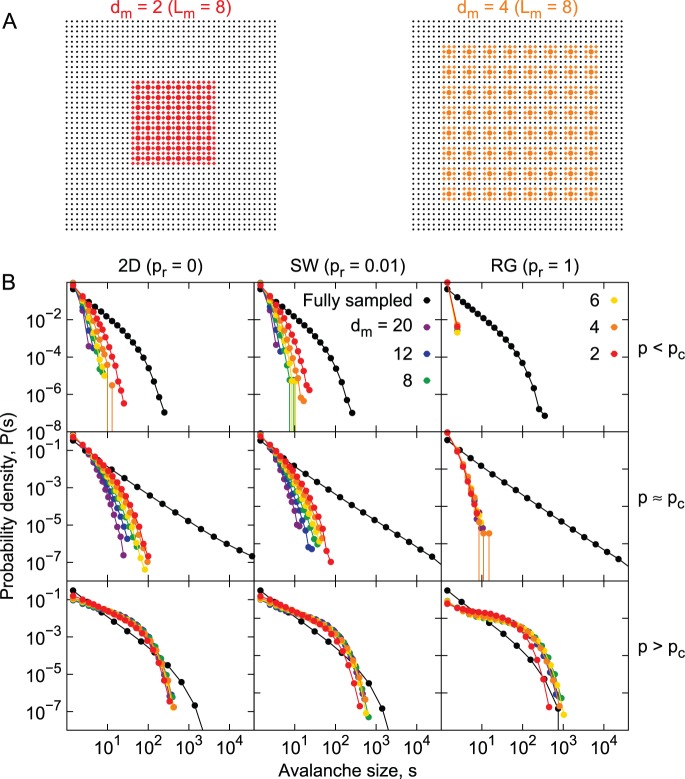
Undersampled size distributions: varying the density of sampled sites. A) Examples of the sampling matrix. Only the colored sites are considered for avalanche measuring. Colored circles indicate virtual electrodes center, with diamond indicating possible sampling sites (see Methods). B) Avalanche size distributions obtained from different underlying topologies (columns) and dynamical regimes (rows), while sampling the systems with a MEA-like configuration. Colors represent different spacing between electrodes of the virtual sampling MEA (black for fully sampled systems).

Next we experimented with keeping the number of electrodes fixed (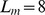) while varying the sampling density via changes in the inter-electrode distance 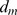 (Fig. 5A). In this case, distributions from the 2D and SW networks behave similarly (left and central columns of Fig. 5B, respectively). Subcritical and critical curves increase their tail gradually as the distance between sampled sites decreases. The supercritical systems, for all topologies, present essentially the same size distributions, independently of the sampling density (Fig. 5B, bottom row). The self-sustained activity, spread through all the network, explains that result. Interestingly, for the random network, all dynamical regimes are similar regarding undersampling: size distributions do not depend on the sampling density (Fig. 5B, right column).

### Comparison with Experimental Data

Avalanche size distributions in the model change due to undersampling, as shown in the previous section. For any combination of the three topologies and three regimes considered in our simple branching-like model, power-law distributions were not observed when the systems were subjected to the same conditions of a typical experiment (tens of electrodes). This, in principle, suggests that, once undersampling is taken into account, this model fails at reproducing spike avalanche size distributions obtained from anesthetized animals and *in vitro* preparations.

However, there is another aspect to consider when comparing model avalanches with experimental ones: the binning procedure. Since neuronal avalanches are defined as a sequence of active bins preceded and followed by empty bins, the temporal bin width plays a fundamental role in avalanche sizes and durations. Clearly, larger bins favor larger avalanches and vice versa. The now standard procedure originally proposed by Beggs and Plenz [1] to address this issue is to calculate the temporal bin width from the data, using the average inter-event interval, or the average interval between consecutive spikes with all neurons considered. We refer to the resulting temporal bin as the optimal bin (see Methods). So far, we have shown size distributions using the natural temporal scale of the model, which is one time step. In order to properly compare model and experimental distributions, however, the same binning procedure should be employed for both.

**Figure 6:**
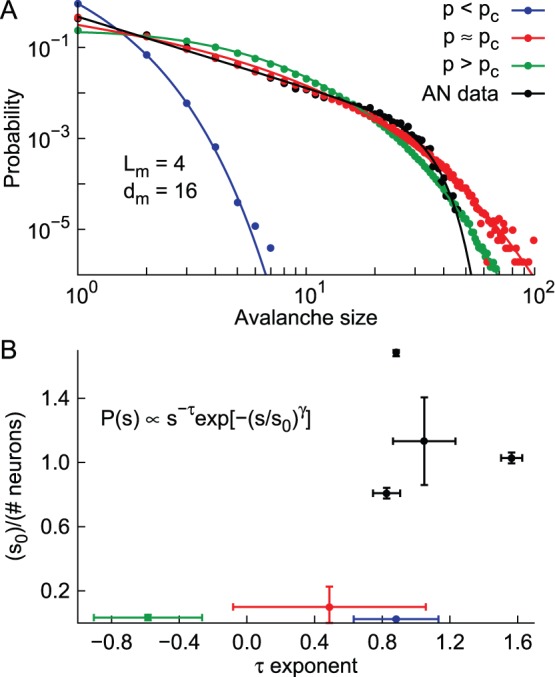
Size distributions for anesthetized animals and undersampled models. A) Avalanche size distributions for the model (subcritical blue, critical red and supercritical green) and an anesthetized rat (black). The MEA employed is the same for all cases (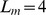, 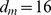). Temporal bins calculated from mean inter-event interval in all cases. Lines represent the best fit of a power law with a sharp cutoff. B) Parameters fitted for each of the model distributions showed in panel A, together with experimental distributions from 4 anesthetized rats. Error bars indicate standard deviation for the values.

We focused on SW networks sampled with 16 electrodes fixed at a distance of 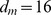 cell bodies. We simulated subcritical, critical and supercritical networks and, for each of these regimes, the optimal bin was calculated and used to obtain the size distributions shown in Fig. 6A. The first observation is that the optimal bin renders distributions which are closer to the full-sampling than those obtained with a bin of one time step (compare with Fig. 4B). Nonetheless, the subcritical (blue circles) and supercritical (green circles) distributions still fail to exhibit a power-law behavior. Furthermore, although the critical distribution (red circles) seems more likely to be well fit by a power law, the expected cutoff for avalanche sizes close to the system size is absent. In fact, very large avalanches (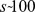) are observed despite the fact that only 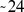 neurons are sampled (average of 1.5 neurons per electrode).

Experimental size distributions from anesthetized rats (AN), on the other hand, are very well fit by a power law with a cutoff (black curve at Fig. 6A). We recorded spiking activity from 4 rats while they were under the effects of ketamine-xylazine anesthesia through MEAs with 32 electrodes and 500 µm spacing (see Methods and Ref. [33]). A subset of 16 electrodes was analyzed, comprising the largest square matrix (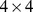) that could be compared to the configuration employed in the model (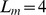, 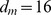).

To quantify the contrast between model and experimental avalanche size distributions, we tried fitting a power law with a sharp cutoff to each of the distributions obtained: 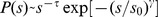, with 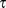, 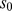 and 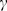 as free parameters. Figure 6A shows that this function fits the data and the simulations in all scenarios. In Fig. 6B, however, we observe that the fitted parameters 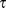 and 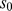, together with their associated errors, are consistently different between model and experimental distributions. While AN data fitting errors are very limited and the cutoff region is in agreement with the number of neurons in each case (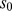 divided by the number of neurons is of order one), the model errors are very large and, more importantly, the values of 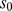 are typically very small. This essentially means that, for the model distributions, the exponential part of the fit is actually dominating the curves, reflecting the fact that they are not well fit by power laws.

## Discussion

We have simulated two-dimensional networks of excitable elements modeled by cellular automata, which have been used in recent works to mimic the propagation of neuronal avalanche [1], [23]–[25]. In particular, we have investigated how undersampling affects spike avalanche size distributions, under different topologies and dynamical regimes. The effects of the investigated topologies can be summarized as follows: two-dimensional and small-world networks are more severely affected by decreasing sampling densities because they have a well defined local neighborhood, in contrast to random graphs, whose size distributions do not change significantly when sampling density decreases.

Undersampled avalanche size distributions obtained from networks with different dynamical regimes have very distinct properties. In subcritical networks, increasing the size of the sampling matrix does not lead to improvement in the distributions (in the sense of bringing it closer to that of a fully sampled system), while increasing the sampling density slowly moves the distributions toward larger avalanches. In supercritical networks, on the other hand, sampling density has no effect on the distributions. However, provided that enough sites are sampled, the behavior of fully sampled supercritical networks can be completely recovered. Critical networks improve with both increasing number of sampled sites as well as increasing sampling density.

Taken together, these results suggest that the dynamical regime of such systems can be retrieved by varying the sampling conditions and comparing the obtained distributions. This could further confirm that the spike avalanches observed in freely-behaving rats [33] come from an underlying critical system. Lognormal size distributions from a critical model (in a simpler version than the one studied here) were shown to be very similar to those found in the experiments. On the other hand, as previously remarked, spike avalanches obtained from *in vitro* preparations and anesthetized rats follow power laws. We have not observed power-law distributions from any undersampled model system, regardless of dynamical regime or network topology. We speculate that the dynamics of *in vitro* and anesthetized systems have additional ingredients which mask the undersampling effects, preserving the power-law size distributions. These ingredients are absent from the models presented here. We have previously shown that, at least for small system sizes, a modified version of the critical model can indeed produce size distributions which seem compatible with power laws even when undersampled [33]. A more complete exploration of the effects of undersampling in that model should be considered for future research.

We conclude that excitable cellular automata in undersampled 2D, SW and RG topologies fail to reproduce spike avalanches from *in vitro* preparations and anesthetized animals. It is important to emphasize that this does not apply to LFP measurements, which is the most common way to record neuronal avalanches. Since LFP captures local currents, it is possible that it can overcome the undersampling effects, thus rendering power-law size distributions. Priesemann and colleagues employed an LFP model to test its robustness against undersampling [36]. In that work, they show that an undersampled LFP model can yield power-law size distributions for avalanches. However, the definition of avalanche size in their undersampled model is not the same as the usual. For instance, there is no binning in the spike time series and information from the underlying (fully sampled) avalanche propagating through the critical system is used to define the end of the undersampled avalanches. The power law becomes an expected result in that scenario. Furthermore, their LFP definition could not be applied to our model, due to the instant transition from inactive to active state in the latter. The hypothesis that LFP could explain power laws observed for neuronal avalanches remains to be investigated.

There are other candidates to reconcile the experimental results with undersampling. It could be a different model, such as the one employed by Poil and colleagues [44], in which neurons are represented by integrate-and-fire units, inhibitory synapses are considered and the transition is from a collectively non-oscillating to an oscillating phase. Or it could be a different topology, such as the one employed by Moretti and Muñoz [45]. The hierarchical modular topologies they propose may sustain robust power laws even with undersampling. These possibilities are beyond the scope of this paper and have yet to be tested. Although undersampling is unavoidable in experiments, it has been generally overlooked in model studies. We propose that it can in fact be a very essential tool to evaluate models when avalanche dynamics are being investigated.

## Materials and Methods

### Ethics Statement

All animal work including housing, surgical and recording procedures were in strict accordance with the National Institutes of Health guidelines, and the Duke University Institutional Animal Care and Use Committee, and was approved by the Edmond and Lily Safra International Institute of Neuroscience of Natal Committee for Ethics in Animal Experimentation (permit # 04/2009).

### Cellular Automaton Model

We have employed a two-dimensional network of excitable cellular automata. In this model, each site 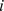 (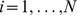; 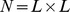) cyclically goes through its 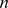 states: 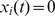 if the 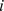-th site is quiescent at time 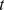; 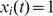 if it is excited; 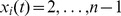 if it is refractory. The model rules are:

- A quiescent site at time 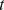 becomes excited at time 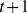 if any of its pre-synaptic neighbors is excited at time 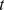 and transmits successfully, each independently with probability 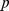;
- An excited site at time 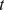 becomes refractory at time 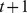 and subsequently runs through the refractory states until it is back to quiescence: 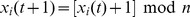 (deterministic dynamics for refractory period).

The network topology is built in a two-step process. Firstly, for each site 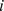, 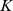 post-synaptic sites are drawn according to an exponential probability distribution of distance between sites, 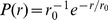 (with 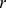 measured in lattice units), and a uniform distribution for the angle between sites, 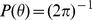. Each synapse has a probability 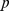 of transmitting a spike. More than one synapse between the same pre- and post-synaptic sites are allowed (in this case increasing the likelihood that a spike is propagated from pre- to post-synaptic sites). Secondly, each synapse has a probability 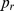 of being rewired and a new post-synaptic site is randomly chosen from all the sites in the network.

The boundaries of the networks are open. The parameters used, their meaning and values are listed in Table 1.

**Table 1:** Parameters of the model.

Parameter	meaning	value
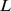	linear size of the lattice	100–300
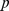	transmission probability	[0,1]
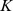	# outgoing synapses per neuron	16
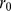	characteristic radius of synaptic reach (cell bodies)	5
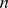	# states of the CA	4
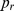	rewiring probability	0, 0.01, 1

Parameters employed in the cellular automaton model.

### Response Curves and Avalanches

In order to measure the density of active sites 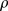, each model neuron is independently driven by a Poissonian stimulus with rate 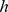. We then average the number of spikes per time step for a long time (at least 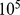 time steps) after waiting for a transient time (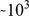 time steps), necessary for the network activity to become stable. In the statistical physics literature, 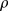 is known as the order parameter for this model. To find the critical point we varied 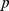 until a power-law behavior for 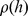, with the expected critical exponent [2], was obtained.

In order to study avalanche propagation we start with a completely quiescent network and fire the central site. Then we wait until it dies out (except for supercritical systems, in which we stop when 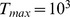 time steps is reached). The size of an avalanche is defined as the number of spikes during its propagation. For better visualization, avalanche size distributions were obtained through logarithmic binning. In other words, we calculate the probability density of observing an avalanche in a range of values. This range is chosen so that points in the x-axis of the log-log plots are equally spaced.

### Undersampling

In order to investigate undersampling effects in the model we implemented a sampling matrix mimicking the experimental MEAs. The sampling matrix is a square 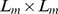 array (centered in the network) of “virtual electrodes”, with a distance 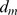 between electrodes. Each one of these electrodes can capture the activity from up to 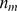 of the 9 closest sites. The actual number of sampled sites in each electrode is drawn from a uniforme distribution between 0 and 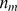. The parameters used can be seen in Table 2.

**Table 2:** Parameters of the sampling matrix.

Parameter	meaning	value
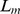	linear size of the matrix	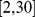
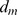	inter-electrode distance (cell bodies)	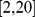
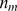	maximum number of neurons detectable at each electrode	3

Parameters employed in the sampling matrix.

Unless otherwise stated, all calculations employed a temporal bin of one time step of the model. When comparing with experiments, the average inter-event interval (IEI) was employed. The IEI corresponds to the time difference between consecutive spikes of the network, regardless of the identity of the neuron. Due to the infinite separation of time scales in the model, we calculated the inter-event interval only during the propagation of the avalanches.

### Experiments

A total of 4 adult male Long-Evans rats (300–350 g) were used for electrophysiological recordings. Multielectrode arrays (35 µm tungsten wires, 32 wires per array, 500 µm spacing, 1 M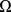 at 1 kHz) were surgically positioned within the primary somatosensory (S1) and visual (V1) neocortices on the left hemisphere. Positioning was verified during or after surgery by spontaneous and evoked activity profiles, and confirmed by post-mortem histological analysis [33], [46].

One to five weeks after a 10-day recovery period, animals were recorded during anesthesia (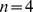). From each electrode spike times from up to 4 nearby neurons were sampled at 40 kHz, whereas LFP were sampled at 500 Hz. Multiple action potentials (spikes) and LFPs were simultaneously recorded using a 96-channel Multi-Neuron Acquisition Processor (MAP, Plexon Inc, Dallas, TX), as previously described [33], [46]. Briefly, single-unit recordings were performed using a software package for real-time supervised spike sorting (SortClient 2002, Plexon Inc, Dallas, TX). Spike sorting was based on waveform shape differences, peak-to-peak spike amplitudes plotted in principal component space, characteristic inter-spike-interval distributions, and a maximum 1% of spike collisions assuming a refractory period of 1 ms. Candidate spikes with signal-to-noise ratio lower than 2.5 were discarded. A waveform-tracking technique with periodic template adjustment was employed for the continuous recording of individual units over time. In order to ensure the stability of individual neurons throughout the experiment, waveform shape and single neuron clustering in principal component space were evaluated using graphical routines (WaveTracker software, Plexon, Dallas, TX). Ellipsoids were calculated by the cluster mean and 3 standard deviations corresponding to two-dimensional projections of the first and second principal components over consecutive 30 min data recordings. Strict superimposition of waveform ellipsoids indicated units that remained stable throughout the recording session and were therefore used for analyses, while units with nonstationary waveforms were discarded. Animals received a single intramuscular administration of ketamine chlorhydrate (100 mg/kg) and xylazine (8 mg/kg), plus a subcutaneous injection of atropine sulfate (0.04 mg/kg) to prevent breathing problems. Anesthetized animals were placed inside a dark chamber and recorded for 4–6 hours, until they recovered waking behavior.
